# Venous thromboembolism in hospitalized patients with COVID-19: clinical and laboratory characteristics at three tertiary care centers in a state capital in the Eastern Amazon

**DOI:** 10.1590/1677-5449.202500762

**Published:** 2026-07-20

**Authors:** Ester Almeida Carneiro Rodrigues da Silva, Adson Kevin Cunha Negidio, Tiago de Oliveira Vieira, Thais Pinheiro Almeida dos Santos, Luiz Felipe Santiago Bittencourt, Paulo Martins Toscano

**Affiliations:** 1 Universidade Federal do Pará – UFPA, Belém, PA, Brasil.; 2 Instituição Estácio IDOMED-Castanhal, Castanhal, PA, Brasil.; 3 Hospital Municipal de Castanhal Dra. Maria Laise Moreira Pereira Lima, Castanhal, PA, Brasil.; 4 Universidade do Estado do Pará – UEPA, Belém, PA, Brasil.; 5 Instituto Evandro Chagas, Belém, PA, Brasil.

**Keywords:** biomarkers, COVID-19, pulmonary embolism, deep venous thrombosis

## Abstract

**Background:**

To date, the COVID-19 pandemic constitutes the greatest health care challenge of the century. Its central pathophysiology involves an infectious respiratory disease caused by the SARS-CoV-2 virus with potential in the most severe cases to provoke cardiovascular complications, including venous thromboembolism (VTE).

**Objectives:**

This study describes the clinical and laboratory characteristics of hospitalized patients with VTE confirmed during a COVID-19 infection.

**Methods:**

This is an observational, longitudinal, and retrospective analytical study based on review of the medical records of patients admitted to three tertiary centers in the capital of Pará state, Brazil, with VTE diagnosed by imaging. Epidemiological and laboratory data were collected retrospectively at each health center, covering the period starting when the first case of COVID-19 was recorded and ending in July 2021.

**Results:**

VTE cases were recorded in 1.30% of medical records, with percentages varying from 0.60% to 2.25% at the different centers. Mean age, C-reactive protein, D-dimer, body mass index, and length of hospital stay were 46.35 years, 143.23 mg/L, 4.12 µg/mL, 27.65 kg/m^2^, and 50.18 days, respectively. No significant correlations (p < 0.05) were observed between presence of VTE and any of variables studied.

**Conclusions:**

There appeared to be associations between some of the variables tested, but it was not possible to confirm any causal relationships. It is possible that epidemiological associations between COVID-19 and VTE could be measured and better explained by possible confounding factors, such as simultaneous coinfections, preexisting health conditions, and others.

## INTRODUCTION

In December 2019, COVID-19 first broke out in Wuhan, Hubei, China. The disease is caused by the novel SARS-CoV-2. It spread rapidly, provoking substantial mortality and becoming the most severe global health crisis of the century.^1^ On March 11, 2020, the COVID-19 outbreak was declared a pandemic by the World Health Organization (WHO), spreading to more than 100 countries worldwide in just 5 months, while the prevalence of cases and deaths continued to rise.^2^ As of March 31, 2024, more than 774 million confirmed cases had been notified globally, with more than 7 million deaths.^3^

The medical and scientific community has been aware that SARS-CoV-2 infection can provoke extra-respiratory manifestations since the start of the COVID-19 pandemic. Endotheliitis, hypercoagulability, and hypofibrinolysis were identified in patients with COVID-19 as secondary responses to endothelial dysfunction, dysfunction of the renin-angiotensin-aldosterone system, elevated von Willebrand factor activation, and cytokine “storm”.^4,5^ Some studies propose that this activation could be directly associated with the elevated frequency of thromboembolic events (TEs) that are particularly observed in critical cases, and even in the presence of pharmaco-prophylaxis.^6-9^

An inflammatory and prothrombotic state can be identified and measured in these patients using biomarkers such as D-dimer (DD) and C-reactive protein (CRP). The hypercoagulability observed in severe forms of COVID-19 provokes an increase in serum DD levels, reflecting exacerbated fibrinolysis.^10^

Although elevated DD has a low positive predictive value, it can suggest the presence of deep venous thrombosis (DVT) and pulmonary embolism (PE), which are the most common thromboembolic events in patients with COVID-19.^10-14^

In addition to serum DD levels, another effective way to identify inflammation is by quantifying serum CRP. CRP has been well-documented in TEs, since is a protein present in the acute phase of inflammation, and high serum levels appear to be associated with COVID-19 mortality.^15^ To date, there is a paucity of studies that describe and discuss the many nuances of the health conditions associated with occurrence of TEs in patients admitted for COVID-19 in the Eastern Brazilian Amazon.

Moreover, in contrast to other Brazilian populations, the Amazonian population has cultural and social elements that are associated with higher prevalence rates of comorbidities, increasing its susceptibility to severe COVID-19.^16,17^

Despite the ample international literature on TEs in patients with COVID-19, the majority of published studies were conducted at centers with good availability of diagnostic and therapeutic resources. In Brazil, and particularly in the Eastern Amazon region, there are persistent structural inequalities affecting access to healthcare, diagnostic limitations, and logistic challenges imposed by geographic factors, all of which can impact both recognition and management of these conditions. Confirming this scenario, Costa et al.^18^ analyzed hospital admissions for TEs in Brazil from 2019 to 2023, finding that the impact of the pandemic was not equal in the different regions of the country, highlighting that scarcity of health professionals, limited access to imaging, and a prioritization of care related to COVID-19 were impeding diagnosis and treatment of severe conditions such as TEs. To date, there is a paucity of multicenter studies describing the clinical and laboratory profile of these patients in the context of Brazil’s North administrative region.

Therefore, the objective of this study was to describe the clinical and laboratory profile of patients admitted with COVID-19 who had TEs while in the following hospitals: Fundação Santa Casa de Misericórdia do Pará (FSCMPA), Hospital Universitário João de Barros Barreto (HUJBB), and Hospital Nossa Senhora de Guadalupe (HNSG).

## METHODS

### Study design and setting

This retrospective, observational, analytical study was conducted at tertiary hospitals in Belém, the capital city of the Brazilian state of Pará, comprising the following institutions: FSCMPA, HUJBB, and HNSG.

### Sample

The study population comprised patients admitted with COVID-19 who were diagnosed with DVT and/or PE on the basis of imaging studies during the periods starting with the first case registered at each center and running to July 30, 2021. Patients who developed DVT and/or PE but were not diagnosed with COVID-19 during the same hospital stay were excluded from the sample, as were patients whose medical records did not provide at least of the two laboratory variables of interest (DD or CRP).

This is thus a convenience sample comprising all patients with COVID-19 who were admitted to one of the three participating centers during the study period and had a diagnosis of venous thromboembolism (VTE) confirmed by imaging. Since this is a retrospective study surveying all eligible cases, no sample size calculation was conducted in advance. Thus, the 17 cases included constitute the totality of all records available that met the inclusion criteria. Therefore, concepts of margin of error and ideal sample size are not applicable, since they presuppose randomized selection of participants. The results observed thus reflect the behavior of the analyzed dataset, supporting identification of relevant associations and tendencies, with no intention to make statistical inferences to the general population.

### Data collection

Data were analyzed from patients with a diagnosis of COVID-19, International Classification Of Diseases, 10th revision (ICD-10) codes B34.2 (Coronavirus infection, unspecified) and B97.2 (Coronavirus as the cause of diseases classified elsewhere). The variables collected for the study were: hospital, age, sex, date of admission, date of end of hospital stay, date admitted to intensive care unit (ICU), date of end of ICU stay, body mass index (BMI), DVT (Yes/No), PE (Yes/No), death (Yes/No), date of death, serum DD level, date DD level tested, DD measurement unit, serum CRP level, date CRP level tested, and CRP measurement unit.

**Figure 1:**
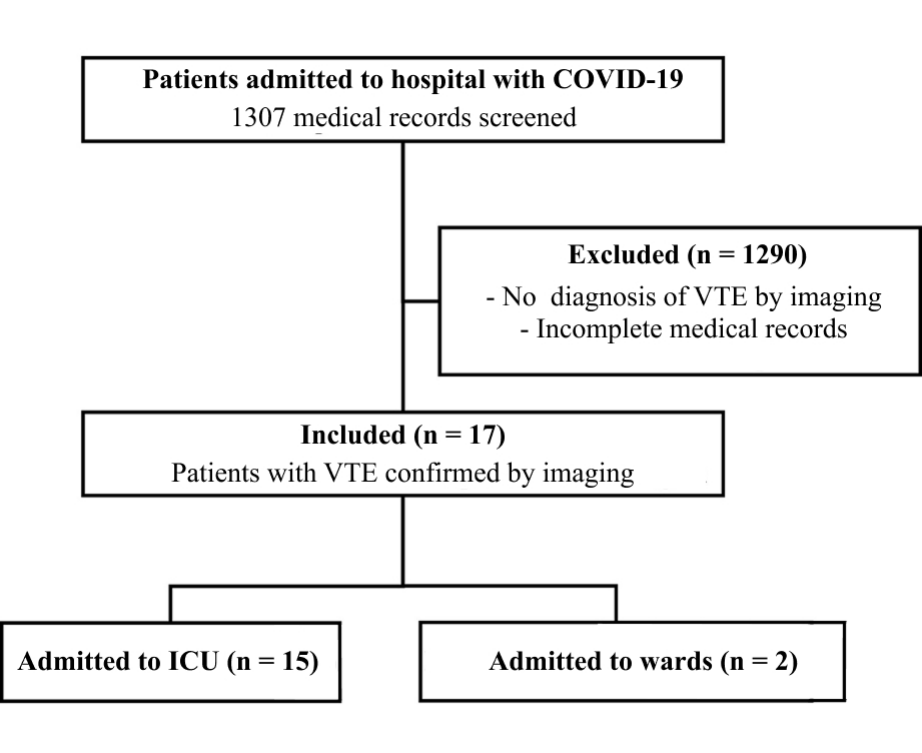
Flow diagram illustrating selection of hospitalized patients with COVID-19 for the study sample. VTE = venous thromboembolism; ICU = intensive care unit.

Data on variables were collected from electronic and physical patient records stored by the FSCMPA Medical Records Administration (GAME) and the HUJBB Care Regulation Department (SRAS), while only electronic patient records were used for the HNSG patients. Serum DD and CRP levels were obtained from the outsourced laboratory service for FSCMPA and HNSG and using HUJBB’s Lab software. Finally, a detailed flow diagram compliant with the Strengthening the Reporting of Observational Studies in Epidemiology (STROBE) guidelines was produced illustrating the total number of medical records, application of exclusion criteria, and the final sample analyzed (Figure 1).

### Research ethics committee (REC)

The study was conducted under the institutional responsibility of the Instituto de Ciências da Saúde (ICS), part of Universidade Federal do Pará, with the participation of FSCMPA, HUJBB, and HNSG. The study was approved by the RECs at the participating institutions: ICS, CAAE: 54741321.0.0000.0018, substantiated opinion No. 5.197.343; FSCMPA, CAAE: 54741321.0.3002.5171, substantiated opinion No. 5.315.889; and HUJBB, CAAE: 54741321.0.3001.0017, substantiated opinion No. 5.286.482. The HNSG does not have a REC, so authorization for data collection was granted by the board of directors, with the approval of the coordinating institution’s REC (ICS/ Universidade Federal do Pará) and no need for submission to any other committee. All researchers involved signed Consent to Terms of Database Use for FSCMPA, HUJBB, and HNSG.

### Statistical analysis

Data were analyzed using R, version 4.1.2. The sample characteristics were described in terms of mean, median, minimum, maximum, amplitude, and standard deviation for the variables age, DD, CRP, BMI, total number of days in hospital, and number of days in the ICU. For the variable age, these measures were calculated by sex and for the entire sample, while for all other variables, measures were calculated separately for VTE type (DVT or PE) and for the entire sample.

Absolute and relative frequencies were calculated for the qualitative variables hospital, type of TEs, sex, diabetes mellitus, and systemic arterial hypertension.

Considering the limitations of the sample, non-parametric statistical tests were chosen, because these are inference methods that do not assume that the data conform to a specific type of distribution, such as normal, and are generally based on ranks or frequencies, rather than the original absolute values, making them more appropriate for cases with small samples, offering greater robustness, although with less statistical power if parametric conditions are met.

For quantitative variables, Spearman’s rank-order correlation coefficient was used as test of significance, as implemented in R, using Algorithm AS 89 developed by Best and Roberts^19^ to determine the p-value. The Wilcoxon-Mann Whitney test was used to analyze associations between qualitative and quantitative variables, also as implemented in R, which uses the procedure presented by Bauer^20^ to determine the p-value. For all tests, the cutoff adopted for rejection of the null hypothesis was 5% (𝛼 = 0.05). Additionally, nonparametric tests we used to test the variables analyzed for normality.

## RESULTS

**Figure 2:**
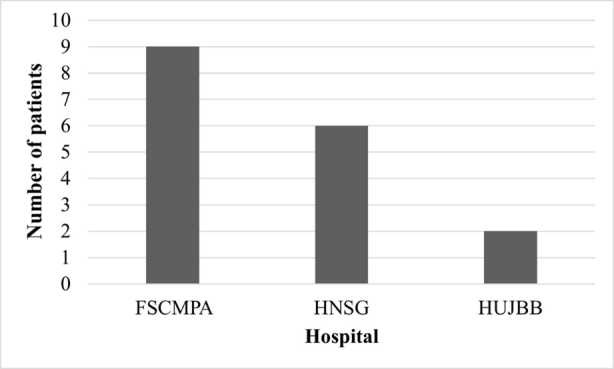
Number of patients per hospital. FSCMPA = Fundação Santa Casa de Misericórdia do Pará; HNSG = Hospital Nossa Senhora de Guadalupe; HUJBB = Hospital Universitário João de Barros Barreto.

Of 1,307 medical records screened, 17 (1.30%) were included in the study sample because they reported VTE confirmed by imaging (15 in the ICU and 2 on wards). Figure 1 shows a flow diagram illustrating selection of medical records for the study sample. There was significant variability in the number of cases of VTE at each hospital: 0.60% at HUJBB (2 DVTs in 321 records), 1.25% at FSCMPA (9 DVTs in 720 records), and 2.25% at HNSG (5 PEs and 1 DVT in 266 records). Figure 2 illustrates the distribution by hospital of patients who had TEs.

With regard to patient sex, the majority of the patients who underwent TEs were female, totaling 11 patients (64.71%), while 6 (35.29%) were male. There was great variability with relation to patient age, ranging from 2 to 85 years. Table 1 lists the main statistical results related to patient age, broken down by sex.

**Table 1:** Statistical measures of patient age, by sex.

**Sex**	**Minimum**	**Maximum**	**Mean**	**Median**	**Range**	**Standard deviation**
Female	2.00	82.00	46.00	60.00	80.00	31.50
Male	12.00	85.00	45.00	48.50	73.00	26.16
Total	2.00	85.00	46.00	54.00	83.00	28.89

With regard to patients’ serum DD levels, it was observed that the two groups of patients, those with DVT and those with PE, had relatively similar means and medians, but that the variability in DD levels was considerably larger among patients with DVT than among those who had PE, ranging from 0.36 to 14.45 µg/mL. The patients’ CRP levels exhibited considerable variability, ranging from 5.90 to 621.50 mg/L. There was also a high degree of divergence between the levels of patients with DVT and those with PE, who had very different means, medians, and standard deviations. Since some patients had serum CRP and DD levels assayed several times, the statistical measures were calculated using the highest serum CRP and DD levels observed for each patient (Table 2).

**Table 2:** Statistical measures of the variables D-dimer level, C-reactive protein level, body mass index, and days in hospital, by type of thromboembolism.

**Type of thromboembolism**	**Minimum**	**Maximum**	**Mean**	**Median**	**Range**	**Standard deviation**
DD level						
DVT	0.36	14.45	4.50	3.28	14.09	4.21
PE	1.22	4.00	3.27	3.93	2.78	1.37
Total	0.36	14.45	4.12	3.45	14.09	3.56
CRP level						
DVT	5.90	621.50	156.70	128.30	615.60	166.34
PE	16.50	218.20	113.50	113.00	201.70	78.09
Total	5.90	621.50	143.20	122.10	615.60	140.86
BMI						
DVT	18.50	44.59	24.44	20.87	26.09	10.04
PE	22.70	50.25	31.51	30.08	27.55	10.99
Total	18.50	50.25	27.65	23.28	31.75	10.60
Days in hospital						
DVT	20.00	124.00	55.50	50.00	104.00	33.05
PE	9.00	88.00	37.40	25.00	79.00	32.07
Total	9.00	124.00	50.18	41.00	115.00	32.87

DD = D-dimer; CRP = C-reactive protein; BMI = body mass index; DVT = deep vein thrombosis; PE = pulmonary embolism.

With respect to patients’ BMI, it was observed that patients with DVT and those with PE had relatively similar means and medians, but that the variation in patient BMI was very high, ranging from underweight patients (<18.50 kg/m^2^) to patients with grade III obesity (≥40.00 kg/m^2^).

There was also considerable variability in the number of days spent in hospital, ranging from 9 to 124 days, with a mean hospital stay among the patients who had DVT that was approximately 20 days longer than for those who had PE (Table 2).

The Spearman correlation coefficients for analysis of quantitative variables did not reveal any significant associations (p > 0.05) for any of the variables (maximum DD and CRP serum levels, BMI, days in hospital, and number of days in the ICU), whether for the analysis based on maximum serum DD levels (Table 3) or for maximum CRP levels (Table 4).

**Table 3:** Result of the nonparametric test for correlations between maximum D-dimer level and all other variables.

**Variable**	**Spearman Coefficient**	**95% CI**	**p**
Maximum CRP level	0.081	(-0.517; 0.626)	0.803
BMI	0.110	(-0.524; 0.666)	0.763
Days in hospital	-0.100	(-0.637; 0.503)	0.734
Days in ICU	-0.070	(-0.619; 0.525)	0.828

95% CI = 95% confidence interval; BMI = body mass index; CRP = C-reactive protein; ICU = intensive care unit.

**Table 4:** Results of the nonparametric test for correlations between maximum C-reactive protein level and all other variables.

**Variable**	**Spearman Coefficient**	**95% CI**	**p**
Maximum DD level	0.081	(-0.517; 0.626)	0.803
BMI	-0.097	(-0.659; 0.534)	0.789
Days in hospital	0.130	(-0.430; 0.618)	0.633
Days in ICU	0.300	(-0.274; 0.717)	0.229

95% CI = 95% confidence interval; DD = D-dimer; BMI = body mass index; ICU = intensive care unit.

The Wilcoxon-Mann Whitney test was used to test for associations between serum DD and serum CRP levels in terms of the outcome discharge or death. For this test, the null hypothesis tested was that the serum DD and CRP levels of patients who were discharged would have the same distribution as the serum DD and CRP levels of those who died. The coefficient for the statistical test for DD was 12.00 (p = 0.921) and the coefficient for analysis of CRP was 13.00 (p = 0.439). On the basis of these results, with a significance level of 5.00%, it was concluded that the null hypothesis cannot be rejected, i.e., there is no statistical evidence that patients’ serum levels of either DD or CRP influenced the outcome discharge or death, since both groups had the same distribution.

Nevertheless, it was observed that only patients who developed DVT died (3 patients), while 9 patients who had DVT and 5 who had PE were discharged.

## DISCUSSION

In this series of 17 cases, patient age ranged from 2 to 85 years. The incidence of VTE observed was 1.30%, which could be considered low compared with reports in the literature. The majority of studies have reported much higher and considerably varied incidence or prevalence rates. Among patients studied by Martinot et al.^21^ and Al-Samkari et al.,^22^ 4.00% and 4.80%, respectively, exhibited VTE confirmed by imaging. However, datasets such as those of Thondapu et al.^23^ and Liang et al.^24^ revealed percentages of 24.60% and 20.00%, respectively, of hospitalized patients with VTE. In contrast, a Japanese study observed a very low rate of VTE (0.80%).^25^

Analyses of data from 2019 to 2022 conducted by Saliba et al.^26^ found a high proportion of occurrence of lower limb DVT among patients with COVID-19. That study also reported that the majority of diagnoses were made using color Doppler ultrasonography. Similarly, Rocha and Sanchez^27^ published a review article of publications from 2020 to 2023 showing that patients hospitalized for COVID-19 were at increased risk of development of TEs, especially those admitted to an ICU. In this respect, the low frequency of cases in the present study could be indicative of limitations related to availability of appropriate diagnostic methods at the participating centers, which could have contributed to under-notification of TEs.

The differences in our results and the disparities in numbers of cases at the different centers could be because of characteristics intrinsic to the services themselves. While this is a multicenter study of 1,307 medical records from patients with confirmed COVID-19, the absence of PE cases at HUJBB and FSCMPA may be linked to their lack of pulmonary tomography angiography (CTA), which is the diagnostic tool used for confirmation of this type of VTE, implying that it is possible that there is a certain incongruence in the results.

This limitation was present at both the public health service providers in the study, but not at the only private center. This center, HNSG, has better infrastructure, with greater CTA availability, and is also a center of excellence for intensive care. VTE may very often go undiagnosed because of its variable clinical presentation, which can even be asymptomatic, considering that the majority of deaths from VTE occur without clinical confirmation of the condition.^28^

Although there was insufficient evidence to confirm a statistically significant correlation, it was notable that there was a higher rate of VTE among women, with a total of 11 (64.71%) women in the study sample, although a previous meta-analysis reported a different result, with male patients with COVID-19 being more likely to have VTE (69.00%).^29^ A univariate analysis also concluded that males were more associated with VTE.^23^ In contrast, a meta-analysis by Lobbes et al.^30^ did not find any association between risk of VTE and male sex.

In this study, the mean age observed was 46.35 years, lower than the mean age of patients with COVID-19 and VTE reported in the literature (64.50 years), demonstrating that VTE is more common among individuals with COVID-19 and advanced age.^29^ Another study found that 100% of the patients who developed VTE were over the age of 40 years, with 57.14% of them aged from 40 to 80 years, which is similar to our findings, where seven patients (41.17%) were in this age group.^24^ It should be noted that there were four patients less than 18 years old in our study, all at FSCMPA, which is a referral center for pediatric treatment in the state capital. Thus, including data from a center that receives pediatric referrals in a small sample may have caused mean and median age to shift artificially. Nonetheless, in line with the findings of this study, the literature shows there is no relationship between presence of VTE and age.^30^

The mean serum DD level observed was 4.12 µg/mL, with the majority of patients’ levels being concentrated in the range from 3.00 to 4.50 µg/mL. These data are in agreement with the literature, which shows that elevated serum DD levels, even during initial presentation, are predictive of thromboembolic complications including VTE during hospital admissions (DD > 2.50 µg/mL).^22^ In our sample, highly variable values were observed, primarily among patients who developed DVT, possibly because the majority of these patients were hospitalized at centers that did not have CTA. Moreover, two patients with considerably higher serum CRP levels than the others had, respectively, septic arthritis and an infected sacral ulcer, concurrently with the COVID-19 infection.

In a study by Smilowitz et al.,^31^ the median CRP level among 2,601 hospitalized patients with COVID-19 was 108 mg/L and CRP concentrations exceeding the median value were associated with VTE and mortality, when compared with CRP below the median. In the present study, the mean CRP level was 143.23 mg/L, with a median of 122.10 mg/L, which is a higher median value than observed by Smilowitz et al.,^31^ since the study only describes the subpopulation of patients with VTE. Additional markers at initial presentation that were predictive of TEs during hospital admissions included CRP > 100 mg/L.^22^ The results of that meta-analysis are consistent with cohort studies that found positive associations between elevated serum CRP levels and disease severity.^13^

A meta-analysis by Wu et al.^29^ found that mean BMI among patients with COVID-19 who had VTE at admission was 27.22 kg/m^2^ in a population with BMI ranging from 25.70 to 28.75 kg/m^2^. In another study, patients with VTE had a mean BMI of 26.90 kg/m^2^.^25^ Those findings corroborates the findings of the present study, which observed a mean BMI of 27.65 kg/m^2^, with median of 23.28 kg/m^2^. Notwithstanding, other studies have concluded that there is no relationship between risk of VTE and BMI or obesity.^30^

With respect to the number of days in hospital, a high degree of variability was observed in the sample, with a range of 9 to 124 days, mean of 50.18 days, and median of 41.00 days. In line with these findings, the literature reports moderate certainty on the association between risk of VTE and length of hospital stay.^30^

The present study has limitations inherent to its retrospective design, small sample, and basis in review of medical records, which causes dependence on the quality and completeness of the records available. The small and non-probabilistic sample limits the study’s statistical power and prevents robust inferences to the general population. Additionally, the heterogeneous nature of the infrastructure at the different participating centers, especially with respect to access to diagnostic methods such as pulmonary CTA, could have contributed to underdiagnosis and variability between the centers. The lack of temporal standardization of testing of laboratory markers and the possibility of unmeasured confounding factors should also be considered in interpretation of the results. Therefore, the findings are primarily a reflection of the cases recorded and do not necessarily reflect the true magnitude of VTE incidence among hospitalized patients with COVID-19 in the context studied.

## CONCLUSION

In this retrospective multicenter study, the frequency of VTE was low among hospitalized patients with COVID-19 and no statistically significant associations were observed between occurrence of VTE and clinical and laboratory variables. These results should be interpreted with caution because of the small sample size, the possibility of underdiagnosis, and the heterogeneous nature of the centers involved. Additionally, the low frequency of events, despite the large number of medical records screened, may have limited identification of more consistent correlations and the large differences in variables such as age and BMI within a small sample could have influenced the results. Further studies with more robust methodologies and greater standardization of data collection and analysis could contribute to better understanding of the magnitude of VTE among hospitalized patients with COVID-19 and of the factors associated with it. As such, the findings of this study should primarily be interpreted as a description of the profile of the cases identified at the participating centers.
