# Supplementary material for: Venous thromboembolism in hospitalized patients with COVID-19: clinical and laboratory characteristics at three tertiary care centers in a state capital in the Eastern Amazon
Source: J Vasc Bras. 2026 Jul 20;25:e20250076. doi: 10.1590/1677-5449.202500762 (PMC13441218; doi:10.1590/1677-5449.202500762)
Supplement: Translation

# Translation: Tromboembolismo venoso em pacientes hospitalizados com covid-19: características clínicas e laboratoriais em três centros de assistência terciária de uma capital da Amazônia Oriental

**DOI:** 10.1590/1677-5449.202500761

**Authors:** Ester Almeida Carneiro Rodrigues da Silva, Adson Kevin Cunha Negidio, Tiago de Oliveira Vieira, Thais Pinheiro Almeida dos Santos, Luiz Felipe Santiago Bittencourt, Paulo Martins Toscano

**Affiliations:** 1 Universidade Federal do Pará – UFPA, Belém, PA, Brasil.; 2 Instituição Estácio IDOMED-Castanhal, Castanhal, PA, Brasil.; 3 Hospital Municipal de Castanhal Dra. Maria Laise Moreira Pereira Lima, Castanhal, PA, Brasil.; 4 Universidade do Estado do Pará – UEPA, Belém, PA, Brasil.; 5 Instituto Evandro Chagas, Belém, PA, Brasil.

**Keywords:** biomarcadores, covid-19, embolia pulmonar, trombose venosa profunda

## Abstract

**Contexto:**

A pandemia da covid-19 se apresenta, até o momento, como o maior desafio sanitário deste século. A fisiopatologia central implica em uma doença respiratória infecciosa causada pelo SARS-CoV-2 capaz de provocar complicações cardiovasculares nos casos mais graves, incluindo o tromboembolismo venoso (TEV).

**Objetivos:**

Este trabalho visa caracterizar o perfil clínico e laboratorial de pacientes internados com TEV documentados durante a infecção por covid-19.

**Métodos:**

Trata-se de um estudo observacional, longitudinal e retrospectivo de natureza analítica em que foram revisados prontuários de pacientes hospitalizados e diagnosticados por imagem com TEV em três centros médicos de assistência terciária da capital do Pará, Brasil. Dados epidemiológicos e laboratoriais foram coletados retrospectivamente em cada centro de saúde, abrangendo o período desde o primeiro caso de covid-19 registrado até julho de 2021.

**Resultados:**

Foram identificados casos de TEV em 1,30% dos prontuários, variando de 0,60 a 2,25% nos diferentes centros. Os valores médios de idade, proteína C reativa, dímero-D, índice de massa corporal e tempo de internação foram, respectivamente, 46,35 anos, 143,23 mg/L, 4,12 µg/mL, 27,65 kg/m^2^ e 50,18 dias. Não foram encontradas correlações significativas (p < 0,05) entre nenhuma das variáveis estudadas e presença de TEV.

**Conclusões:**

Parece haver alguma associação entre algumas das variáveis testadas, porém sem possibilidade de estabelecimento de relação causal. É possível que as associações epidemiológicas entre covid-19 e TEV possam ser mediadas e mais bem explicadas por eventuais fatores de confusão, como coinfecções simultâneas, condições de saúde prévias, entre outros.

## INTRODUÇÃO

Em dezembro de 2019, surgiu em Wuhan, Hubei, China, a doença por coronavírus 2019 (covid-19) causada pela síndrome respiratória aguda grave por coronavírus-2 (SARS-CoV-2), propagada rapidamente e com mortalidade substancial, que se tornou a mais grave crise sanitária global deste século^1^. Em 11/03/2020, a covid-19 foi declarada pandemia pela Organização Mundial da Saúde (OMS), envolvendo mais de 100 países em todo o mundo em apenas 5 meses, enquanto a prevalência de casos e mortes seguiu aumentando^2^. Até 31/03/2024, foram notificados globalmente mais de 774 milhões de casos confirmados e mais de 7 milhões de mortes^3^.

Desde o início da pandemia de covid-19, a comunidade médica e científica estava ciente das manifestações extra-respiratórias da infecção por SARS-CoV-2. Endotelite, hipercoagulabilidade e hipofibrinólise foram identificadas em pacientes com covid-19 como respostas subsequentes de disfunção endotelial, disfunção do sistema renina-angiotensina aldosterona, ativação aumentada do fator de Von Willebrand e “tempestade” de citocinas^4,5^. Alguns estudos propõem que tal ativação pode estar diretamente associada à elevada frequência de eventos tromboembólicos (ETs) observada especialmente em casos críticos e mesmo quando do uso de farmacoprofilaxia^6-9^.

O estado inflamatório e pró-trombótico desses pacientes pode ser identificado e mensurado por meio de biomarcadores, como dímero-D (DD) e proteína C reativa (PCR). A hipercoagulabilidade observada nas formas graves da covid-19 implica em aumento dos níveis séricos de DD como um reflexo da fibrinólise exacerbada^10^.

Apesar de seu baixo valor preditivo positivo, o DD elevado pode sugerir a presença de trombose venosa profunda (TVP), bem como de tromboembolismo pulmonar (TEP), os ETs mais frequentes em pacientes com covid-19^10-14^.

Além dos níveis séricos de DD, outra forma eficiente de verificação da inflamação ocorre por meio da quantificação sérica da PCR. Como uma proteína de fase aguda da inflamação, a PCR é bem documentada nos ETs, além de haver associação de seus altos níveis séricos com mortalidade na covid-19^15^.

Até o momento, são escassos estudos que descrevam e discutam as diversas nuances das condições médicas associadas à presença de ETs no contexto da Amazônia brasileira Oriental em pacientes internados com covid-19. Além disso, diferentemente de outras populações brasileiras, a população amazônica fundamenta-se em elementos culturais e sociais associados à maior prevalência de comorbidades, aumentando, assim, a suscetibilidade à gravidade da covid-19^16,17^.

Apesar da ampla literatura internacional sobre ETs em pacientes com covid-19, a maioria dos estudos disponíveis foi conduzida em centros com ampla disponibilidade de recursos diagnósticos e terapêuticos. No Brasil, particularmente na região da Amazônia Oriental, persistem desigualdades estruturais no acesso à saúde, limitações diagnósticas e desafios logísticos impostos por fatores geográficos, os quais podem influenciar tanto o reconhecimento quanto o manejo dessas condições. Corroborando esse cenário, Costa et al.^18^, ao analisarem as internações por ETs no Brasil entre 2019 e 2023, evidenciaram o impacto desigual da pandemia nas diferentes regiões do país, destacando que a escassez de profissionais de saúde, a limitação no acesso a exames de imagem e a priorização dos atendimentos relacionados à covid-19 dificultaram o diagnóstico e o tratamento de condições graves, como os ETs. Até o momento, são escassos estudos multicêntricos que descrevam o perfil clínico e laboratorial desses pacientes no contexto regional do Norte.

Nesse sentido, o objetivo deste estudo foi caracterizar o perfil clínico e laboratorial de pacientes internados com covid-19 que apresentaram ETs nos hospitais Fundação Santa Casa de Misericórdia do Pará (FSCMPA), Hospital Universitário João de Barros Barreto (HUJBB) e Hospital Nossa Senhora de Guadalupe (HNSG).

## MÉTODOS

### Desenho e contexto do estudo

Este estudo observacional e retrospectivo de natureza analítica foi realizado nos hospitais de assistência terciária de Belém, PA, abrangendo as instituições FSCMPA, HUJBB e HNSG.

### Amostra

A população da pesquisa incluiu pacientes internados com covid-19 com diagnóstico de imagem de TVP e/ou TEP, compreendendo pacientes internados desde o primeiro caso (em cada serviço) até 30/07/2021. Foram excluídos da pesquisa pacientes sem diagnóstico de covid-19 que desenvolveram TVP e/ou TEP na mesma internação e pacientes que não possuíam pelo menos uma das duas variáveis laboratoriais (DD ou PCR) em seu prontuário.

Trata-se de uma amostra de conveniência composta por todos os pacientes hospitalizados com covid-19 nos três centros avaliados que apresentaram diagnóstico confirmado de tromboembolismo venoso (TEV) por método de imagem no período estudado. Por se tratar de um estudo retrospectivo e censitário dos casos elegíveis, não foi realizado cálculo de tamanho amostral prévio. Assim, os 17 casos incluídos representam a totalidade dos registros disponíveis que preencheram os critérios de inclusão. Por esse motivo, não se aplicam os conceitos de margem de erro ou de tamanho ideal de amostra que pressupõem seleção aleatória dos participantes. Assim, os resultados obtidos refletem o comportamento do conjunto analisado, permitindo identificar associações e tendências relevantes, sem pretensão de inferência estatística para a população geral.

### Coleta de dados

Foram analisados os dados de pacientes com diagnóstico de covid-19, com o código da Classificação Internacional de Doenças, 10ª revisão (CID-10) de infecção por coronavírus de localização não especificada (B34.2) e de coronavírus como causa de doenças classificadas em outros capítulos (B97.2). As variáveis incluídas no estudo foram hospital, idade, sexo, data da internação, data de encerramento da internação, data de entrada na unidade de terapia intensiva (UTI), data de saída da UTI, índice de massa corporal (IMC), TVP (sim/não), TEP (sim/não), óbito (sim/não), data do óbito, níveis séricos de DD, data de verificação dos níveis de DD, unidade de medida do DD, níveis séricos da PCR, data de verificação dos níveis da PCR e unidade de medida da PCR.

A coleta das variáveis foi realizada nos prontuários eletrônicos e físicos na gerência de arquivo médico da FSCMPA e no setor de regulação assistencial do HUJBB, sendo utilizados apenas prontuários eletrônicos no serviço do HNSG. Os níveis séricos de DD e de PCR foram recolhidos do site do laboratório terceirizado que atende o serviço da FSCMPA e do HNSG, e, para a coleta no HUJBB, foi utilizado o programa Lab. Por fim, em conformidade com as diretrizes do *Strengthening the Reporting of Observational Studies in Epidemiology* (STROBE) *Statement*, foi construído um fluxograma detalhado (Figura 1) com o número total de prontuários avaliados, os critérios de exclusão e a amostra final analisada.

**Figura 1:** Fluxograma de seleção dos pacientes hospitalizados com covid-19 incluídos no estudo. TEV = tromboembolismo venoso; UTI = unidade de terapia intensiva.

### Comissão de ética em pesquisa (CEP)

A pesquisa foi conduzida sob responsabilidade institucional do Instituto de Ciências da Saúde (ICS) da Universidade Federal do Pará, com a participação das instituições FSCMPA, HUJBB e HNSG. O estudo foi aprovado pelos CEPs das respectivas instituições: ICS (CAAE: 54741321.0.0000.0018, parecer consubstanciado nº 5.197.343); FSCMPA (CAAE: 54741321.0.3002.5171, parecer consubstanciado nº 5.315.889); HUJBB (CAAE: 54741321.0.3001.0017, parecer consubstanciado nº 5.286.482). O HNSG não possui CEP próprio, sendo a coleta de dados autorizada pela diretoria da instituição, com a aprovação do CEP da instituição proponente (ICS/Universidade Federal do Pará), não sendo necessária a submissão a outro comitê. Todos os envolvidos assinaram o Termo de Consentimento de Uso de Banco de Dados da FSCMPA, do HUJBB e do HNSG.

### Análise estatística

A análise dos dados foi realizada com o uso do software R, versão 4.1.2. Para a caracterização da amostra, foram calculados média, mediana, mínimo, máximo, amplitude e desvio-padrão para as variáveis idade, DD, PCR, IMC, dias de internação total e dias de internação em UTI. Para a variável idade, as referidas medidas foram calculadas separadamente por sexo e pelo total de pacientes, ao passo que, para as demais variáveis, as medidas foram calculadas separadamente por tipo de TEV (TVP ou TEP) e pelo total.

Para as variáveis qualitativas hospital de internação, tipo de ETs, sexo, presença de diabetes melito e hipertensão arterial sistêmica, foram calculadas as frequências absolutas e relativas.

Considerando as limitações da amostra, optou-se por utilizar testes estatísticos não paramétricos, pois são métodos de inferência que não exigem que os dados sigam uma distribuição específica, como a normal, baseando-se geralmente em postos ou frequências em vez dos valores originais, sendo mais adequados em situações com amostras pequenas, oferecendo maior robustez, embora com menor poder estatístico quando as condições paramétricas são satisfeitas.

Para as variáveis quantitativas, foi utilizado o teste de significância sobre coeficiente de correlação de postos de Spearman, implementado no software R, o qual utiliza o Algoritmo AS 89, desenvolvido por Best e Roberts^19^, para determinação do valor de p. Já para a análise de associação entre variáveis qualitativas e quantitativas, foi utilizado o teste de Wilcoxon-Mann-Whitney, também implementado no software R, o qual utiliza o procedimento apresentado por Bauer^20^ para determinação do valor de p. Em todos os testes foi adotado o limite de rejeição da hipótese nula de 5% (𝛼 = 0,05). Além disso, também se optou por utilizar testes não paramétricos para a verificação da normalidade das variáveis analisadas.

## RESULTADOS

Do total de 1.307 prontuários revisados, 17 (1,30%) foram incluídos no estudo por apresentarem TEV confirmado por exame de imagem (15 em UTI e 2 em enfermaria). A Figura 1 apresenta o fluxograma de seleção dos prontuários incluídos no estudo. Foi encontrada variabilidade significativa do número de casos detectados de TEV entre cada hospital: HUJBB de 0,60% (2 TVPs de 321), FSCMPA de 1,25% (9 TVPs de 720) e HNSG de 2,25% (5 TEPs e 1 TVP de 266). A distribuição dos pacientes que apresentaram ETs por hospital de internação foi demonstrada na Figura 2.

**Figura 2:** Quantidade de pacientes por hospital. FSCMPA = Fundação Santa Casa de Misericórdia do Pará; HNSG = Hospital Nossa Senhora de Guadalupe; HUJBB = Hospital Universitário João de Barros Barreto.

A maioria dos pacientes que apresentou ETs era do sexo feminino, sendo 11 pacientes (64,71%) do sexo feminino e 6 (35,29%), do sexo masculino. Em relação à idade, pôde-se observar uma variabilidade bem alta, com pacientes a partir de 2 anos até 85 anos. A Tabela 1 apresenta as principais medidas estatísticas sobre a idade dos pacientes distribuídos por sexo.

**Tabela 1:** Medidas estatísticas da idade dos pacientes por sexo.

**Sexo**	**Mínimo**	**Máximo**	**Média**	**Mediana**	**Amplitude**	**Desvio-padrão**
Feminino	2,00	82,00	46,00	60,00	80,00	31,50
Masculino	12,00	85,00	45,00	48,50	73,00	26,16
Total	2,00	85,00	46,00	54,00	83,00	28,89

Em relação aos níveis séricos de DD dos pacientes, observou-se que tanto os pacientes que tiveram TVP quanto os pacientes que tiveram TEP apresentaram médias e medianas relativamente próximas, porém, a variabilidade nos níveis de DD dos pacientes que tiveram TVP foi bem maior que os que tiveram TEP, com amplitude entre 0,36 e 14,45 µg/mL. Em relação aos níveis de PCR dos pacientes, observou-se uma alta variabilidade, com amplitude entre 5,90 e 621,50 mg/L. Verificou-se também uma alta divergência nas medidas dos pacientes que tiveram TVP em relação aos que tiveram TEP, pois esses apresentaram médias, medianas e desvio-padrão bem distantes. Considerando que para alguns pacientes o nível sérico de PCR e DD foram verificados várias vezes durante o período de internação, foram utilizados apenas o maior nível sérico de PCR e de DD observados para cada paciente no cálculo das medidas estatísticas (Tabela 2).

**Tabela 2:** Medidas estatísticas das variáveis do nível de dímero-D e de proteína C reativa, índice de massa corporal e dias de internação por tipo de tromboembolismo.

**Tipo de tromboembolismo**	**Mínimo**	**Máximo**	**Média**	**Mediana**	**Amplitude**	**Desvio- padrão**
Nível de DD						
TVP	0,36	14,45	4,50	3,28	14,09	4,21
TEP	1,22	4,00	3,27	3,93	2,78	1,37
Total	0,36	14,45	4,12	3,45	14,09	3,56
Nível de PCR						
TVP	5,90	621,50	156,70	128,30	615,60	166,34
TEP	16,50	218,20	113,50	113,00	201,70	78,09
Total	5,90	621,50	143,20	122,10	615,60	140,86
IMC						
TVP	18,50	44,59	24,44	20,87	26,09	10,04
TEP	22,70	50,25	31,51	30,08	27,55	10,99
Total	18,50	50,25	27,65	23,28	31,75	10,60
Dias de internação						
TVP	20,00	124,00	55,50	50,00	104,00	33,05
TEP	9,00	88,00	37,40	25,00	79,00	32,07
Total	9,00	124,00	50,18	41,00	115,00	32,87

DD = dímero-D; PCR = proteína C reativa; IMC = índice de massa corporal; TVP = trombose venosa profunda; TEP = tromboembolismo pulmonar.

No que se refere ao IMC dos pacientes, constatou-se que tanto os pacientes que tiveram TVP quanto os que tiveram TEP apresentaram médias e medianas relativamente próximas, porém, a variabilidade no IMC dos pacientes foi bastante alta, com pacientes abaixo do peso (< 18,50 kg/m^2^) e pacientes com obesidade grau III (≥ 40,00 kg/m^2^).

E em relação aos dias de internação dos pacientes, observou-se uma alta variabilidade, com intervalo de 9 a 124 dias de internação. Além disso, a média de internação dos pacientes que tiveram TVP foi de aproximadamente 20 dias a mais que a média dos que tiveram TEP (Tabela 2).

Na análise das variáveis quantitativas, o coeficiente de correlação de Spearman não constatou associação significativa (p > 0,05) em todas as variáveis (níveis séricos máximos de DD e PCR, IMC, dias de internação e dias de internação em UTI), tanto na análise em função dos níveis séricos de DD máximo (Tabela 3) quanto de PCR máximo (Tabela 4).

**Tabela 3:** Resultado do teste não paramétrico de correlação entre o nível máximo de dímero-D em relação às demais variáveis.

**Variável**	**Coeficiente de Spearman**	**IC95%**	**p**
Nível máximo de PCR	0,081	(-0,517; 0,626)	0,803
IMC	0,110	(-0,524; 0,666)	0,763
Dias de internação	-0,100	(-0,637; 0,503)	0,734
Dias de internação em UTI	-0,070	(-0,619; 0,525)	0,828

IC95% = intervalo de confiança de 95%; IMC = índice de massa corporal; PCR = proteína C reativa; UTI = unidade de terapia intensiva.

**Tabela 4:** Resultados do teste não paramétrico de correlação entre o nível máximo de proteína C reativa em relação às demais variáveis.

**Variável**	**Coeficiente de Spearman**	**IC95%**	**p**
Nível máximo de DD	0,081	(-0,517; 0,626)	0,803
IMC	-0,097	(-0,659; 0,534)	0,789
Dias de internação	0,130	(-0,430; 0,618)	0,633
Dias de internação em UTI	0,300	(-0,274; 0,717)	0,229

IC95% = intervalo de confiança de 95%; DD = dímero-D; IMC = índice de massa corporal; UTI = unidade de terapia intensiva.

Para verificar a associação entre os níveis séricos de DD e de PCR em relação ao desfecho de alta ou óbito, foi utilizado o teste de Wilcoxon-Mann-Whitney. Para tal, considerou-se a hipótese nula de que os níveis séricos de DD e de PCR dos pacientes que tiveram alta seguiu a mesma distribuição dos níveis séricos de DD e PCR dos que vieram a óbito. Assim, no teste estatístico para o DD, o coeficiente foi de 12,00 (p = 0,921), e para a análise do coeficiente para o PCR foi de 13,00 (p = 0,439). Com esses resultados, concluiu-se, com nível de significância de 5%, que a hipótese nula não pode ser rejeitada, ou seja, que não há indícios estatísticos de que os níveis séricos, tanto de DD quanto de PCR, dos pacientes influenciam no desfecho de alta ou óbito, visto que ambos seguem a mesma distribuição.

Além disso, foi possível verificar que apenas pacientes que tiveram TVP vieram a óbito (3 pacientes), ao passo que entre os pacientes com evolução favorável, 9 tiveram TVP e 5 TEP.

## DISCUSSÃO

Na série de 17 casos, foram identificadas idades que variaram de 2 a 85 anos. A incidência encontrada de TEV foi de 1,30%, a qual pode ser considerada baixa quando comparada à reportada na literatura existente. A maioria dos estudos demonstraram incidências ou prevalências mais elevadas e consideravelmente variadas. Entre os pacientes estudados por Martinot et al.^21^ e Al-Samkari et al.^22^, 4 e 4,80% apresentaram TEV confirmada com exame de imagem, respectivamente. No entanto, dados como os apresentados nos estudos de Thondapu et al.^23^ e Liang et al.^24^ constataram valores respectivos de 24,60 e 20,00% de pacientes com TEV no contexto de hospitalização. Em contrapartida, um estudo japonês encontrou uma diminuta taxa de TEV (0,80%)^25^.

Nos levantamentos realizados por Saliba et al.^26^, foi verificada uma grande proporção de ocorrência de TVP de membros inferiores em pacientes com covid-19 entre os anos de 2019 e 2022. O estudo constatou também que, na maioria dos diagnósticos, o método de imagem utilizado foi o eco-Doppler colorido. Em consonância, Rocha e Sanchez^27^, em seu artigo de revisão abrangendo publicações entre os anos de 2020 e 2023, destacaram que pacientes hospitalizados por covid-19 apresentam risco aumentado para desenvolvimento de ETs, especialmente aqueles internados em UTI. Nesse sentido, a baixa frequência de casos na presente pesquisa pode apontar limitações relacionadas à disponibilidade de métodos diagnósticos adequados nos serviços avaliados, o que pode ter contribuído para a subnotificação de ETs.

As diferenças em nossos resultados, bem como a disparidade de casos nos diferentes centros deste estudo, podem ser explicadas por características intrínsecas desses mesmos serviços. Embora este seja um estudo multicêntrico com 1.307 prontuários de pacientes com covid-19 confirmado, a ausência de casos de TEP no HUJBB e na FSCMPA pode estar associada à indisponibilidade da angiotomografia pulmonar computadorizada, que é a ferramenta diagnóstica de confirmação para esse tipo de TEV, implicando na possibilidade de alguma incongruência nos resultados.

Tal limitação se manifesta em dois dos três centros deste estudo, mas não no serviço privado. O único centro privado, o HNSG, dispõe de maior infraestrutura e possui disponibilidade da angiotomografia pulmonar computadorizada, sendo um centro de referência também em cuidados intensivos. O TEV pode muitas vezes não ser diagnosticado, devido ao seu quadro clínico variável, inespecífico ou até mesmo assintomático, tendo em vista que a maioria dos óbitos por TEV ocorre sem a confirmação clínica da condição^28^.

Apesar de não haver indícios suficientes para atestar uma correlação estatística significativa, destaca-se o achado de uma quantidade de TEV superior em mulheres, sendo 11 (64,71%) na amostra pesquisada. Não obstante, uma metanálise apresentou resultado diferente, com pacientes do sexo masculino com covid-19 sendo mais propensos a sofrer TEV (69,00%)^29^. Uma análise univariável também concluiu que o sexo masculino foi o mais associado com TEV^23^. Em contraste, a metanálise de Lobbes et al.^30^ não encontrou associação entre o risco de TEV e o sexo masculino.

Neste estudo, foi observado uma idade média de 46,35 anos, abaixo da média de idade de pacientes com covid-19 e TEV relatada na literatura (64,50 anos), demonstrando que o TEV é mais comum entre indivíduos com covid-19 com idade avançada^29^. Outro estudo encontrou 100% dos pacientes que desenvolveram TEV com mais de 40 anos de idade, estando 57,14% deles entre os 40 e os 80 anos, semelhante aos nossos achados, com sete pacientes (41,17%) dentro da mesma faixa etária^24^. Deve-se ressaltar ainda que nosso estudo possui quatro pacientes com menos de 18 anos de idade, todos da FSCMPA, que é referência de tratamento pediátrico na capital. Assim, a inclusão dos dados de um centro referência em pediatria em uma amostra reduzida pode estar deslocando falsamente a média e mediana das idades. Apesar disso, em consonância com os achados desta pesquisa, a literatura demonstra não haver relação entre presença de TEV e idade^30^.

O nível sérico de DD observado teve média de 4,12 µg/mL, com a maioria dos níveis concentrando-se entre 3,00 e 4,50 µg/mL. Esses dados estão de acordo com a literatura, na qual é demonstrado que níveis séricos de DD elevado, mesmo na apresentação inicial, são preditivos de complicações tromboembólicas durante a hospitalização, incluído TEV (DD > 2,50 µg/mL)^22^. Em nossa amostra é possível observar a presença de valores consideravelmente discrepantes, principalmente entre os pacientes que apresentaram TVP, possivelmente em razão de, nesses casos, os pacientes encontrarem-se, em sua maioria, hospitalizados nos serviços que não dispunham de angiotomografia pulmonar computadorizada. Além disso, dois pacientes com níveis séricos de PCR consideravelmente acima dos demais apresentaram artrite séptica e úlcera sacral infectada simultaneamente à infecção por covid-19.

No trabalho de Smilowitz et al.^31^, com 2.601 pacientes hospitalizados com covid-19, foi obtida mediana de PCR de 108 mg/L, apresentando concentrações de PCR associadas ao TEV e à mortalidade acima do valor mediano, em comparação com PCR abaixo da mediana. Já o nível de PCR no presente estudo teve média de 143,23 mg/L e mediana de 122,10 mg/L, valores de mediana acima dos achados de Smilowitz et al.^31^, visto que o estudo caracteriza apenas a subpopulação de pacientes com TEV. Marcadores adicionais na apresentação inicial preditiva de ETs durante a hospitalização incluíram PCR > 100 mg/L^22^. Os resultados dessa metanálise são consistentes com outros estudos de coorte que associaram positivamente os níveis séricos elevados de PCR com a gravidade da doença^13^.

A metanálise de Wu et al.^29^ encontrou que o IMC médio em pacientes com covid-19 que apresentaram TEV na internação foi de 27,22 kg/m^2^, em uma população com IMC variando de 25,70 a 28,75 kg/m^2^. Em outro estudo, pacientes com TEV apresentaram IMC médio de 26,90 kg/m^225^. Tal achado corrobora o presente trabalho, o qual obteve média de 27,65 kg/m^2^ e mediana de 23,28 kg/m^2^ para IMC. Apesar disso, outros estudos concluíram não haver relação entre o risco de TEV e IMC ou obesidade^30^.

No que se refere aos dias de internação dos pacientes, observa-se uma alta variabilidade da amostra, com intervalo de 9 a 124 dias, média de 50,18 e mediana de 41,00 dias. Em consonância com esses achados, a literatura evidencia certeza moderada para associação entre o risco de TEV e o tempo de permanência na hospitalização^30^.

Assim, este estudo apresenta limitações inerentes ao seu delineamento retrospectivo, à amostra modesta e ao fato de ser baseado em revisão de prontuários, o que implica dependência da qualidade e da completude dos registros disponíveis. A amostra reduzida e não probabilística limita o poder estatístico e impede inferências robustas para a população geral. Além disso, a heterogeneidade estrutural entre os centros participantes, especialmente no que se refere ao acesso a métodos diagnósticos como a angiotomografia pulmonar, pode ter contribuído para o subdiagnóstico e a variabilidade entre os serviços. A ausência de padronização temporal na coleta de marcadores laboratoriais e a possibilidade de fatores de confusão não mensurados também devem ser consideradas na interpretação dos resultados. Assim, os achados refletem principalmente os casos registrados e não necessariamente a real magnitude do TEV em pacientes hospitalizados com covid-19 no cenário estudado.

## CONCLUSÃO

Neste estudo retrospectivo multicêntrico, a frequência de TEV em pacientes hospitalizados com covid-19 foi baixa, e não foram observadas associações estatisticamente significativas entre variáveis clínico-laboratoriais e ocorrência de TEV. Esses resultados devem ser interpretados com cautela, em razão do pequeno tamanho amostral, do possível subdiagnóstico e da heterogeneidade entre os centros. Além disso, a baixa frequência de eventos, apesar do grande número de prontuários revisados, pode ter limitado a identificação de correlações mais consistentes, e diferenças expressivas em variáveis, como idade e IMC, dentro de uma casuística reduzida podem ter influenciado os resultados. Estudos adicionais, com metodologias mais robustas e maior padronização na coleta e na análise dos dados, poderão contribuir para melhor compreensão da magnitude e dos fatores associados ao TEV em pacientes hospitalizados com covid-19. Assim, os achados deste estudo devem ser interpretados principalmente como uma descrição do perfil dos casos identificados nos serviços avaliados.
